# Hospital COVID-19 Burden and Adverse Event Rates

**DOI:** 10.1001/jamanetworkopen.2024.42936

**Published:** 2024-11-04

**Authors:** Mark L. Metersky, David Rodrick, Shih-Yieh Ho, Deron Galusha, Andrea Timashenka, Erin N. Grace, Darryl Marshall, Sheila Eckenrode, Harlan M. Krumholz

**Affiliations:** 1 Division of Pulmonary, Critical Care and Sleep Medicine, University of Connecticut School of Medicine, Farmington; 2 Agency for Healthcare Research and Quality, Department of Health and Human Services, Washington, DC; 3 Center for Outcomes Research and Evaluation, Yale New Haven Hospital, New Haven, Connecticut; 4 Section of Cardiovascular Medicine, Department of Internal Medicine, Yale School of Medicine, New Haven, Connecticut

## Abstract

**Importance:**

The COVID-19 pandemic introduced stresses on hospitals due to the surge in demand for care and to staffing shortages. The implications of these stresses for patient safety are not well understood.

**Objective:**

To assess whether hospital COVID-19 burden was associated with the rate of in-hospital adverse effects (AEs).

**Design, Setting, and Participants:**

This cohort study used data from the Agency for Healthcare Research and Quality’s Quality and Safety Review System, a surveillance system that tracks the frequency of AEs among selected hospital admissions across the US. The study sample included randomly selected Medicare patient admissions to acute care hospitals in the US between September 1, 2020, and June 30, 2022.

**Main Outcomes and Measures:**

The main outcome was the association between frequency of AEs and hospital-specific weekly COVID-19 burden. Observed and risk-adjusted rates of AEs per 1000 admissions were stratified by the weekly hospital-specific COVID-19 burden (daily mean number of COVID-19 inpatients per 100 hospital beds each week), presented as less than the 25th percentile (lowest burden), 25th to 75th percentile (intermediate burden), and greater than the 75th percentile (highest burden). Risk adjustment variables included patient and hospital characteristics.

**Results:**

The study included 40 737 Medicare hospital admissions (4114 patients [10.1%] with COVID-19 and 36 623 [89.9%] without); mean (SD) patient age was 73.8 (12.1) years, 53.8% were female, and the median number of Elixhauser comorbidities was 4 (IQR, 2-5). There were 59.1 (95% CI, 54.5-64.0) AEs per 1000 admissions during weeks with the lowest, 77.0 (95% CI, 73.3-80.9) AEs per 1000 admissions during weeks with intermediate, and 97.4 (95% CI, 91.6-103.7) AEs per 1000 admissions during weeks with the highest COVID-19 burden. Among patients without COVID-19, there were 55.7 (95% CI, 51.1-60.8) AEs per 1000 admissions during weeks with the lowest, 74.0 (95% CI, 70.2-78.1) AEs per 1000 admissions during weeks with intermediate, and 79.3 (95% CI, 73.7-85.3) AEs per 1000 admissions during weeks with the highest COVID-19 burden. A similar pattern was seen among patients with COVID-19. After risk adjustment, the relative risk (RR) for AEs among patients admitted during weeks with high compared with low COVID-19 burden for all patients was 1.23 (95% CI, 1.09-1.39; *P* < .001), with similar results seen in the cohorts with (RR, 1.33; 95% CI, 1.03-1.71; *P* = .03) and without (RR, 1.23; 95% CI, 1.08-1.39; *P* = .002) COVID-19 individually.

**Conclusions and Relevance:**

In this cohort study of hospital admissions among Medicare patients during the COVID-19 pandemic, greater hospital COVID-19 burden was associated with an increased risk of in-hospital AEs among both patients with and without COVID-19. These results illustrate the need for greater hospital resilience and surge capacity to prevent declines in patient safety during surges in demand.

## Key Points

**Question:**

Was hospital COVID-19 burden associated with increased rates of in-hospital adverse events (AEs) during the COVID-19 pandemic?

**Findings:**

In this cohort study of 40 737 hospital admissions among Medicare patients, increased hospital COVID-19 burden was associated with an increased adjusted risk of inpatient AEs among both patients with and without COVID-19.

**Meaning:**

The results of this study suggest a need for greater resilience in hospitals to prevent declines in patient safety and effectiveness of care during increases in demand, such as from pandemics, natural disasters, or other causes.

## Teaser

This cohort study examines whether hospital COVID-19 burden was associated with rates of in-hospital adverse events among Medicare patients during the COVID-19 pandemic.

## Introduction

While hospitalized patients continue to experience adverse events (AEs) at an unacceptable rate, there have been well-documented improvements in patient safety in US hospitals since the publication of the Institute of Medicine’s landmark report, *To Err Is Human: Building a Safer Health System*.^1^ A culture of patient safety has become more ingrained, and this changing culture, accompanied by extensive efforts to improve patient safety, has produced tangible results.^2,3^

The unprecedented strain on hospitals caused by the COVID-19 pandemic and accompanying surges in patients requiring hospitalization and intensive care unit treatment has created concern that recent improvements in hospital patient safety might have been reversed.^4^ Some factors, such as staff burnout and staffing changes (increased use of traveler and per diem staff), may persist beyond the pandemic. Indeed, numerous publications have documented the implications of the COVID-19 pandemic for quality in general^5,6,7^ and patient safety specifically. Hospital-acquired infections, including central line–associated bloodstream infections (CLABSIs) and catheter-associated urinary tract infections (CAUTIs), occurred with increased frequency among 148 hospitals^8^ and among hospitals reporting to the Centers for Disease Control and Prevention’s (CDC) National Healthcare Safety Network (NHSN)^9^ as COVID-19 burden increased. In Taiwan, inpatient falls also increased during the COVID-19 pandemic compared with 2019.^10^ Limitations of many of these studies are that they did not adjust for the high severity of illness associated with COVID-19 hospitalization, investigate the implications for safety among patients who did not have COVID-19, or incorporate hospital-specific COVID-19 results. Thus, some studies might merely have been measuring the impact of the increased severity of illness associated with COVID-19, and studies demonstrating no association between COVID-19 burden and frequency of AEs could have had imprecise estimates of COVID-19 burden. A study of hospital mortality among Medicare patients discharged without a diagnosis of COVID-19 that did adjust for hospital-specific COVID-19 burden found little correlation between mortality and hospital-specific COVID-19 rates.^11^

In this study, we assessed the association between AEs among Medicare patient hospitalizations across the US and the inpatient COVID-19 burden with which the hospital was contending at the time of the AE. We also assessed the association between hospital COVID-19 burden and AEs both for patients without a principal or secondary discharge diagnosis including COVID-19 and those with a discharge diagnosis of COVID-19.

## Methods

### Study Sample

The Yale University institutional review board reviewed the protocol for this cohort study and granted a waiver of informed consent because data were deidentified. We followed the Strengthening the Reporting of Observational Studies in Epidemiology (STROBE) guideline for the reporting of cohort studies.^12^ The study sample was based on data abstracted from inpatient medical records of Medicare patients using the Quality and Safety Review System (QSRS), a medical record abstraction–based national surveillance system developed by the Agency for Healthcare Research and Quality (AHRQ) to track the frequency of adverse events in hospitalized patients,^13^ as well as selected demographic and administrative data abstracted from the medical record by the Clinical Data Abstraction Center.

On an annual basis, hospital samples were selected in a 2-stage process designed by the Centers for Medicare & Medicaid Services in specific categories (strata) to estimate patient safety nationally among the Medicare population. The 5 strata are critical-access hospitals, Indian Health Service (IHS) hospitals, rural Inpatient Prospective Payment System (IPPS) hospitals, targeted IPPS hospitals, and all other acute care hospitals. The IHS hospitals were excluded from this study due to lack of reported COVID-19 patient impact and hospital capacity data.

In the first stage of the sampling process, hospitals were selected using probability proportional to size. In the second stage, 10 records were selected per month by each hospital (unless there were fewer than 10 records, in which case all were selected). The final study sample included a combined sample of admitted Medicare patients (both Medicare Advantage and Fee-for-Service) aged 18 years or older and discharged from hospitals in the period from September 1, 2020, to June 30, 2022. The hospitals in the study were acute care hospitals included in the 4 strata.

COVID-19 Reported Patient Impact and Hospital Capacity by Facility data from September 1, 2020, to June 30, 2022, were obtained from the US Department of Health & Human Services,^14^ which provided facility-level data for hospital utilization aggregated on a moving weekly basis during the pandemic. For this analysis, the weekly hospital COVID-19 burden was defined as the daily mean number of adult patients hospitalized with laboratory-confirmed COVID-19 in a given 7-day period per 100 hospital beds (obtained from the 2019 American Hospital Association database). We then used the week of the admission date in the QSRS data to link the hospital’s weekly COVID-19 burden to the clinical outcomes of all Medicare patients admitted to the same facility during the same 7-day period (using the date of admission to assign the patient to a given week).

### Patient and Hospital Characteristics

Patient characteristics, abstracted from medical records, included demographics (age, sex, and race and ethnicity). We included race and ethnicity in our risk-adjustment models because prior work by our group has demonstrated race-related disparities in the risk of in-hospital AEs.^15^ Categories were Hispanic, non-Hispanic Black (hereafter, Black), non-Hispanic White (hereafter, White), and other (included American Indian or Alaska Native, Asian, Native Hawaiian or Other Pacific Islander, and multiracial) or unknown. We calculated the individual Elixhauser-specific comorbidities^16^ for each patient using the *International Statistical Classification of Diseases and Related Health Problems, Tenth Revision* diagnosis codes and created an aggregated number. To adjust for the underlying medical conditions, we used the Clinical Classification Software Refined for the *International Statistical Classification of Diseases and Related Health Problems, Tenth Revision* developed by the AHRQ,^17^ which collapses more than 70 000 individual diagnosis codes into 530 clinically meaningful categories (Clinical Classifications Software [CCS] categories) across 21 body systems. We further limited to the CCS categories with at least 10 AEs and kept the ones that had a *P* < .20 cutoff. Hospital characteristics were obtained from the 2019 American Hospital Association’s Annual Survey Databases and included size (number of beds), teaching status (teaching vs nonteaching), Joint Commission (JC) certification status (yes or no), and capability to perform coronary artery bypass graft surgery (yes or no) and percutaneous coronary intervention (yes or no), as used in prior publications by our group.^2,3^

### Outcome and COVID-19 Burden

Thirty-two in-hospital AE measures based on relevant data from 9 of 11 QSRS modules were selected, as shown in the eBox in Supplement 1. Specific hospital-acquired infections, medication-related AEs, pressure ulcers, falls, and several postprocedural events were the most common of these events captured. The human abstractors using the QSRS rely on documentation recorded in medical records to complete their abstractions. The definitions and algorithms used in the QSRS are consistent with those used by the AHRQ Common Formats for Surveillance and other measures, such as those associated with the CDC’s NHSN.^18^

Using the calculated COVID-19 burden, we classified each patient admission into 1 of 3 categories: (1) low COVID-19 burden, if the rate was less than the 25th percentile of the overall measured burdens at the time of admission; (2) high, if the rate was greater than the 75th percentile of the overall measured burdens; and (3) intermediate, if otherwise. Our primary outcome was a composite outcome of the 32 measures: the number of AEs per 1000 discharges for each of the COVID-19 burden categories.

### Statistical Analysis

We created 3 cohorts: the total sample, patients discharged with a principal or secondary diagnosis of COVID-19 (*International Statistical Classification of Diseases, Tenth Revision, Clinical Modification* code U071), and those discharged without. We performed descriptive analyses to compare patient and hospital characteristics across the 3 cohorts and the 3 COVID-19 burden categories. To assess whether the COVID-19 burden was associated with patients’ risk of AEs, we used the COVID-19 burden multiplied by 10 as a continuous variable and fit mixed-effects models with a Poisson link function. This allowed us to observe the outcome of every 10% increase in hospital COVID-19 burden. Patient clustering within hospitals was accounted for using generalized estimating equations. To assess whether the COVID-19 burden was associated with patients’ risk of AEs, we used the 2 highest COVID-19 burden categories (intermediate and high) as dummy variables and fit mixed-effects models with a Poisson link function. Patient clustering within hospitals was accounted for using generalized estimating equations. Additionally, we fit the same model using the COVID-19 burden multiplied by 10 as a continuous variable. This allowed us to observe the outcomes of every 10% increase in hospital COVID-19 burden.

To account for differences in patient characteristics and severity of illness, the models were adjusted for the patients’ age, sex, race and ethnicity, Elixhauser comorbidities, payer status, admission urgency, admission source, and CCS codes. To account for regional differences in care, all models included an indicator for US Census region. The final model also included the following hospital characteristics: teaching status, JC accreditation, percutaneous coronary intervention capability, coronary artery bypass grafting capability, and number of beds (<50, 50-99, 100-199, 200-399, or ≥400). Analyses were conducted using SAS, version 9.4 (SAS Institute Inc). Two-sided statistical significance was set at *P* < .05 for all analyses.

## Results

We included data from 40 737 hospitalizations to 1210 unique hospitals in our analyses. Table 1 shows patient and hospital characteristics stratified by COVID-19 burden. Patients had a mean (SD) age of 73.8 (12.1) years; 53.8% of hospitalizations were among female patients and 46.2% were among males; and 11.7% were among Black patients, 3.5% among Hispanic patients, 79.8% among White patients, and 5.0% among patients of other or unknown race and ethnicity. The median number of Elixhauser comorbidities was 4 (IQR, 2-5). A total of 4114 hospitalizations (10.1%) were among patients with a principle or secondary diagnosis of COVID-19 and 36 623 (89.9%) were among patients without a diagnosis of COVID-19. Patients’ demographic characteristics differed little based on hospital COVID-19 burden, but there were fewer elective admissions and more emergency admissions in hospitals with higher COVID-19 burden. Hospital characteristics varied based on COVID-19 burden.

**Table 1.** Patient and Hospital Characteristics by COVID-19 Burden^**a**^

Characteristic	Participants, No. %			
	All admissions (N = 40 737)	COVID-19 burden, percentile^b^		
		<25th (n = 10 174)	25th-75th (n = 20 372)	>75th (n = 10 191)
**Patient characteristics**				
COVID-19 status				
No COVID-19	36 623 (89.9)	9933 (97.6)	18 806 (92.3)	7884 (77.4)
COVID-19	4114 (10.1)	241 (2.4)	1566 (7.7)	2307 (22.6)
Age, mean (SD), y	73.8 (12.1)	74.1 (12.2)	73.7 (12.1)	73.8 (11.8)
Age group, y				
<65	6552 (16.1)	1616 (15.9)	3344 (16.4)	1592 (15.6)
65-74	14 143 (34.7)	3410 (33.5)	7099 (34.9)	3634 (35.7)
75-84	12 479 (30.6)	3115 (30.6)	6238 (30.6)	3126 (30.7)
≥85	7563 (18.6)	2033 (20.0)	3691 (18.1)	1839 (18.1)
Sex				
Female	21 920 (53.8)	5568 (54.7)	10 916 (53.6)	5436 (53.3)
Male	18 817 (46.2)	4606 (45.3)	9456 (46.4)	4755 (46.7)
Race and ethnicity				
Hispanic	1423 (3.5)	276 (2.7)	736 (3.6)	411 (4.0)
Non-Hispanic Black	4749 (11.7)	1014 (10.0)	2518 (12.4)	1217 (11.9)
Non-Hispanic White	32 523 (79.8)	8390 (82.5)	16 065 (78.9)	8068 (79.2)
Other or unknown^c^	2042 (5.0)	494 (4.9)	1053 (5.2)	495 (4.9)
Elixhauser comorbidities, median (IQR), No.	4 (2-5)	4 (2-5)	4 (2-5)	4 (2-5)
Payer status				
Medicare Fee-for-Service	19 395 (47.6)	5029 (49.4)	9601 (47.1)	4765 (46.8)
Medicare Advantage	15 751 (38.7)	3705 (36.4)	8036 (39.5)	4010 (39.4)
Other or unknown	5591 (13.7)	1440 (14.2)	2735 (13.4)	1416 (13.9)
Admission urgency				
Elective	4011 (9.9)	1138 (11.2)	2128 (10.5)	745 (7.3)
Emergency	32 189 (79.0)	7793 (76.6)	15 945 (78.3)	8451 (82.9)
Urgent	3171 (7.8)	875 (8.6)	1615 (7.9)	681 (6.7)
Other or unknown	1366 (3.4)	368 (3.6)	684 (3.4)	314 (3.1)
Admission source				
Community	34 866 (85.6)	8673 (85.3)	17 368 (85.3)	8825 (86.6)
Skilled nursing facility	2017 (5.0)	519 (5.1)	994 (4.9)	504 (4.9)
Transfer from another hospital	2052 (5.0)	466 (4.6)	1159 (5.7)	427 (4.2)
Other or unknown	1802 (4.4)	516 (5.1)	851 (4.2)	435 (4.3)
**Hospital characteristics**				
US region				
Midwest	10 743 (26.4)	3080 (30.3)	5087 (25.0)	2576 (25.3)
Northeast	6900 (16.9)	1832 (18.0)	3673 (18.0)	1395 (13.7)
South	15 420 (37.9)	3466 (34.1)	7893 (38.7)	4061 (39.9)
West	7674 (18.8)	1796 (17.7)	3719 (18.3)	2159 (21.2)
Size, beds, No.				
<50	8109 (19.9)	3243 (31.9)	2789 (13.7)	2077 (20.4)
50-99	3869 (9.5)	1300 (12.8)	1705 (8.4)	864 (8.5)
100-199	7811 (19.2)	1819 (17.9)	3725 (18.3)	2267 (22.3)
200-399	11 092 (27.2)	1995 (19.6)	6131 (30.1)	2966 (29.1)
≥400	9856 (24.2)	1817 (17.9)	6022 (29.6)	2017 (19.8)
Median (IQR)	207 (76-387)	124 (25-314)	257 (114-451)	189 (75-357)
Large teaching	5663 (13.9)	980 (9.6)	3613 (17.7)	1070 (10.5)
JC accredited	30 675 (75.3)	6488 (63.8)	16 097 (79.0)	8090 (79.4)
PCI capability	21 202 (52.1)	3709 (36.5)	11 895 (58.4)	5598 (54.9)
CABG capability	16 008 (39.3)	2779 (27.3)	9202 (45.2)	4027 (39.5)
Hospital length of stay, d				
Mean (SD)	5.5 (5.6)	4.7 (4.8)	5.6 (5.6)	6 (6.2)
Median (IQR)	4 (2-7)	3 (2-6)	4 (2-7)	4 (2-7)
In-hospital mortality	1803 (4.4)	295 (2.9)	859 (4.2)	649 (6.4)

Abbreviations: CABG, coronary artery bypass surgery; JC, Joint Commission; PCI, percutaneous coronary intervention.

^a^ COVID-19 burden was defined as the daily mean number of inpatients with COVID-19 per 100 hospital beds each week.

^b^ Ranges for the percentiles were as follows: <25 percentile, 0 to 1.586; 25th to 75th percentile, 1.587 to 10.330; and >75th percentile, 10.331 to 99.048.

^c^ Includes American Indian or Alaska Native, Asian, Native Hawaiian or Other Pacific Islander, and multiracial.

There were 59.1 (95% CI, 54.5-64.0) AEs per 1000 admissions during weeks with the lowest, 77.0 (95% CI, 73.3-80.9) AEs per 1000 admissions during weeks with intermediate, and 97.4 (95% CI, 91.6-103.7) AEs per 1000 admissions during weeks with the highest COVID-19 burden. Among patients without COVID-19, there were 55.7 (95% CI, 51.1-60.8) AEs per 1000 admissions during weeks with the lowest, 74.0 (95% CI, 70.2-78.1) AEs per 1000 admissions during weeks with intermediate, and 79.3 (95% CI, 73.7-85.3) AEs per 1000 admissions during weeks with the highest COVID-19 burden. A similar pattern was seen among patients with COVID-19. The frequency of AEs captured is shown in eTable 1 in Supplement 1. There were only 15 AEs of hospital-acquired COVID-19.

**Figure 1.**
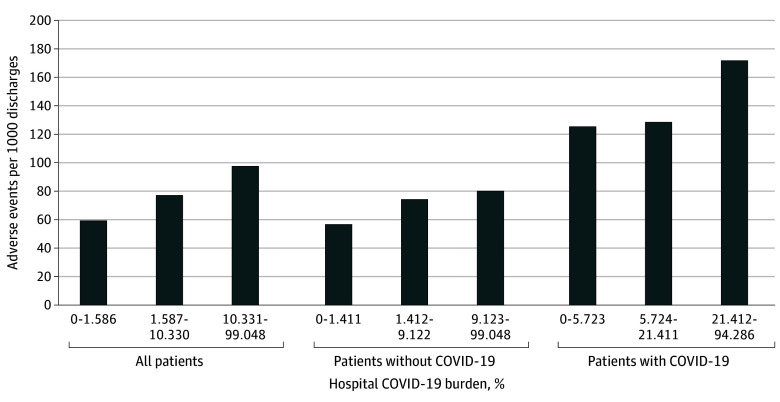
Overall Observed Adverse Events per 1000 Discharges, by Hospital COVID-19 Burden COVID-19 burden was defined as the daily mean number of inpatients with COVID-19 each week per 100 hospital beds.

The observed rates of AEs as a function of COVID-19 burden are presented in Figure 1. An increasing rate of AEs was found as hospital COVID-19 burden increased for patients with and without COVID-19 and for the entire study population. Table 2 shows the unadjusted and risk-adjusted relative risk (RR) of AEs associated with hospital COVID-19 burden. Patients admitted to hospitals with a high, but not intermediate, COVID-19 burden had significantly higher adjusted AE rates for all patients (RR, 1.23; 95% CI, 1.09-1.39; *P* < .001), with a similar pattern for the subgroups without (RR, 1.23; 95% CI, 1.08-1.39; *P* = .002) and with (RR, 1.33; 95% CI, 1.03-1.71; *P* = .03) COVID-19. The associations between COVID-19 burden and AEs analyzed with COVID-19 burden as a continuous variable are presented in eTable 2 in Supplement 1. There was a significant adjusted association between COVID-19 burden and AE rates for the entire population (RR, 1.09; 95% CI, 1.05-1.13; *P* < .001) and the subpopulations without (RR, 1.08; 95% CI, 1.03-1.14; *P* < .001) and with (RR, 1.11; 95% CI, 1.03-1.18; *P* = .003) COVID-19. Data for specific types of AEs are presented in eTable 2 in Supplement 1.

**Table 2.** Risk of Adverse Events Associated With COVID-19 Burden, per 1000 Admissions^**a**^

COVID-19 burden	Unadjusted RR (95% CI)	*P* value	Adjusted RR (95% CI)^b^	*P* value
**Total admissions (N = 40 737)**				
Intermediate vs low COVID-19 burden	1.27 (1.14-1.43)	<.001	1.06 (0.95-1.18)	.32
High vs low COVID-19 burden	1.62 (1.44-1.83)	<.001	1.23 (1.09-1.39)	<.001
**Patients without COVID-19 (n = 36 623)**				
Intermediate vs low COVID-19 burden	1.30 (1.15-1.46)	<.001	1.10 (0.98-1.24)	.11
High vs low COVID-19 burden	1.40 (1.23-1.59)	<.001	1.23 (1.08-1.39)	.002
**Patients with COVID-19 (n = 4114)**				
Intermediate vs low COVID-19 burden	1.02 (0.80-1.31)	.86	0.96 (0.74-1.23)	.72
High vs low COVID-19 burden	1.37 (1.05-1.78)	.02	1.33 (1.03-1.71)	.03

Abbreviation: RR, relative risk.

^a^ COVID-19 burden was defined as the daily mean number of inpatients with COVID-19 per 100 hospital beds each week. Low burden was less than the 25th percentile (0-1.586); intermediate, 25th to 75th percentile (1.587-10.330); and high, greater than the 75th percentile (10.331-99.048).

^b^ Adjusted for patient age, sex, race and ethnicity, admission source, admission urgency, payer source, COVID-19 admission, Elixhauser comorbidities, and Clinical Classification Software diagnosis code; hospital characteristics; and US census region.

**Figure 2.**
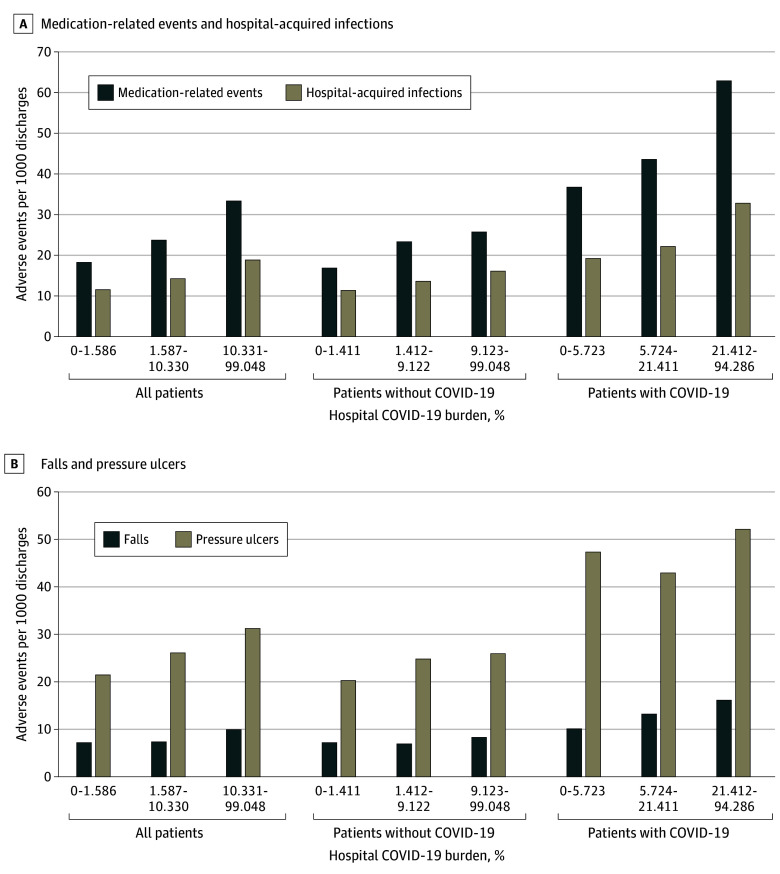
Observed Medication-Related Adverse Events, Hospital-Acquired Infections, Falls, and Pressure Ulcers per 1000 Discharges, by Hospital COVID-19 Burden COVID-19 burden was defined as the daily mean number of inpatients with COVID-19 each week per 100 hospital beds.

We also analyzed the association of hospital COVID-19 burden with specific types of AEs. In Figure 2A, the incidence of observed AE rates by hospital COVID-19 burden is shown for hospital-acquired infections and medication-related AEs, both of which were more frequent as COVID-19 burden increased. Similarly, as shown in Figure 2B, increased incidence of pressure ulcers and fall rates were associated with increasing hospital COVID-19 burden except for pressure ulcer rates in patients with COVID-19, which were lower in hospitals with intermediate than with low COVID-19 burden. Table 3 shows the relative risk of these AEs before and after risk adjustment. Both before and after risk adjustment, the association between high COVID-19 burden and the risk of these AEs varied among the subpopulations; there was increased unadjusted risk among high-burden hospitals for each of these types of events among the entire population, but after adjustment among the entire population, the increased risk remained statistically significant for only medication-related events. In analyses with hospital COVID-19 burden as a continuous variable, COVID-19 burden was associated with medication-related events and with hospital-acquired infections among all patients, patients without COVID-19, and patients with COVID-19. For pressure ulcers and falls, there were also associations except in the COVID-19 group (eTable 2 in Supplement 1).

**Table 3.** Risk, per 1000 Admissions, of Medication-Related Adverse Events, Hospital-Acquired Infections, and Falls or Pressure Ulcers Associated With COVID-19 Burden^a^

Adverse event	All admissions (N = 40 737)				Non–COVID-19 admissions (n = 36 623)				COVID-19 admissions (n = 4114)			
	Unadjusted RR (95% CI)	*P* value	Adjusted RR (95% CI)^b^	*P* value	Unadjusted RR (95% CI)	*P* value	Adjusted RR (95% CI)^b^	*P* value	Unadjusted RR (95% CI)	*P* value	Adjusted RR (95% CI)^b^	*P* value
**Medication-related adverse events**												
Intermediate vs low COVID-19 burden	1.31 (1.08-1.59)	.006	1.03 (0.84-1.25)	.80	1.41 (1.15-1.74)	.001	1.13 (0.91-1.39)	.27	1.19 (0.81-1.77)	.38	1.08 (0.73-1.62)	.69
High vs low COVID-19 burden	1.89 (1.56-2.28)	<.001	1.32 (1.08-1.61)	.007	1.57 (1.26-1.94)	<.001	1.27 (1.02-1.58)	.03	1.75 (1.16-2.63)	.007	1.58 (1.05-2.37)	.03
**Hospital-acquired infections**												
Intermediate vs low COVID-19 burden	1.26 (0.99-1.61)	.06	1.01 (0.79-1.29)	.91	1.22 (0.94-1.58)	.13	0.99 (0.77-1.29)	.96	1.16 (0.63-2.12)	.63	1.25 (0.71-2.21)	.44
High vs low COVID-19 burden	1.71 (1.31-2.23)	<.001	1.30 (0.99-1.71)	.06	1.47 (1.11-1.96)	.007	1.26 (0.95-1.68)	.11	1.76 (0.94-3.29)	.08	2.06 (1.15-3.68)	.02
**Falls or pressure ulcers**												
Intermediate vs low COVID-19 burden	1.18 (1.02-1.38)	.03	1.00 (0.86-1.17)	.99	1.17 (1.00-1.37)	.054	1.04 (0.88-1.22)	.65	0.97 (0.71-1.34)	.88	0.88 (0.63-1.22)	.44
High vs low COVID-19 burden	1.49 (1.27-1.74)	<.001	1.15 (0.97-1.35)	.11	1.27 (1.06-1.52)	.009	1.16 (0.97-1.40)	.10	1.2 (0.84-1.71)	.33	1.14 (0.8-1.61)	.47

Abbreviation: RR, relative risk.

^a^ COVID-19 burden was defined as the daily mean number of COVID-19 inpatients each week per 100 hospital beds.

^b^ Adjusted for patient age, sex, race and ethnicity, admission source, admission urgency, payer source, COVID-19 admission, individual Elixhauser comorbidities, and Clinical Classification Software diagnosis code; hospital characteristics; and US census region.

In addition, to assess whether there could be a threshold of hospital COVID-19 burden associated with increased risk of AE rates, we graphed observed AE rates by decile of hospital COVID-19 burden in the eFigure in Supplement 1. Among patients with COVID-19, the increased AE rate appeared only at the highest hospital COVID-19 burdens, whereas for patients without COVID-19, the largest increase in AE rates occurred as the hospital COVID-19 burden increased from the lowest to the second decile of burden.

## Discussion

The COVID-19 pandemic introduced unprecedented demands on hospitals and their staff, including a surge in the volume of critically ill patients, burden related to isolation protocols, and staff shortages due to illness and other reasons. In this analysis using data from the QSRS, we found that the incidence of AEs occurring among hospitalized patients was associated with the weekly hospital-specific COVID-19 burden from September 2020 through June 2022. Furthermore, this association was seen among patients hospitalized for any reason, including COVID-19, and among patients who did not have COVID-19.

Our study adds to the literature by being the largest and most comprehensive assessment, to our knowledge, of the association of patient safety events with COVID-19 burden at the hospital level. Prior studies have also found an increase in AEs among hospitalized patients during the COVID-19 pandemic,^6,19,20,21^ and their findings complement those of our study. The Leapfrog Group reported that hospital-acquired infections increased to a 5-year high during the pandemic, with the mean CLABSI standard infection ratio (SIR) increased by 60%, the mean methicillin-resistant *Staphylococcus aureus* SIR increased by 37%, and the mean CAUTI SIR increased by 19% compared with the period immediately prior to the COVID-19 pandemic.^19^ These data are concordant with data on hospital-acquired infections reported by the CDC.^6^ In Canada, one health system reported no change in patient safety incident rates coinciding with the onset of the pandemic other than a 75% increase in falls.^20^ Few studies have provided data on patients without COVID-19, although in France, there was increased mortality among non–intensive care unit surgical patients in hospitals with high COVID-19 exposure during 2020.^21^

Our research extends the insights from prior studies^6,19,20,21^ by linking hospital-specific patient AEs with weekly hospital-specific COVID-19 burden as opposed to using national COVID-19 rates, which does not allow adjustment for the variations in COVID-19 rates among different parts of the country. Comparing prepandemic and pandemic data results in a similar lack of granularity and increases the likelihood of temporal trends unrelated to COVID-19 affecting AE rates. In addition, we demonstrated that AE rates increased even among patients without COVID-19, removing the possibility that inadequate risk adjustment among patients with COVID-19—for example, due to their severity of illness—could have affected the validity of the results.

Our results suggest that the significant improvement seen in hospital safety over recent years^2,3^ is fragile and was not resilient to the stress of the pandemic. A surge in patient numbers and/or acuity levels or a staffing shortage—whether related to local, regional, or national events—can adversely affect patient safety, and undoubtedly such events will continue to happen. These realities point to the need for hospitals and health systems to implement systems that function reliably under stress. There is also a need to undertake planning to address future imbalances between patient care needs and staff availability that may occur during patient surges.^22^ Such planning could include a process to quickly implement creative staffing models to address shortages. Prospective governmental planning to allow flexibility in meeting regulatory requirements could also help alleviate staffing shortages, but both of these responses could also increase patient risk if staff are asked to provide care that they lack the skills to perform safely and effectively. There is also evidence that hospital system–level or region-level coordination can facilitate patient load balancing.^23^ Efforts to address stress and burnout among hospital staff to minimize attrition at a time when staffing shortages are most acute might also be useful.^24^

### Limitations

This study has several limitations. Our analysis was limited to the Medicare population, so it might not be applicable to a younger population. AEs were determined by medical record abstraction, so events that were not well documented might have been missed. Documentation practices might have been different during times of high COVID-19-burden, but if so, it seems likely that documentation would have been less complete at these times, resulting in AEs being less likely to be detected during times of high COVID-19 burden. Thus, one would not expect this factor to contribute to the finding of higher AE rates during the pandemic. We adjusted for changes in the reason for hospital admission, as the hospitalized patient population without COVID-19 was likely different during times of high COVID-19 burden due to cancelations of elective surgery and the well-documented tendency of patients to attempt to avoid hospitalization when COVID-19 rates were high.^25^ Our adjustment might not have fully captured these differences in patient characteristics.

One of the AEs collected by the QSRS is hospital-acquired COVID-19, which could be directly affected by COVID-19 rates; however, there were only 15 of these during the entire study period, so any implications for the overall AE rate was negligible. We included patients with a principal or secondary diagnosis of COVID-19, which may have resulted in an underestimation of the impact of COVID-19 burden, as safety among patients with incidentally detected COVID-19 admitted for other reasons might not have been affected as much as among patients with severe COVID-19. As with all observational studies, causation cannot be proven, but the association between hospital-specific COVID-19 rates and AE rates, without another credible theory to explain the results, strongly supports causality.

## Conclusions

This cohort study found a statistically significant increase in AE rates in hospitalized Medicare patients during periods of high COVID-19 burden. This increase was seen in both patients with and without COVID-19. These results illustrate the need for greater resilience, including systems of care that promote patient safety and planning for surge capacity in hospitals to prevent declines in patient safety and effectiveness of care during increases in demand, such as from pandemics, natural disasters, or other causes.

## Notes

Supplement 1. **eBox.** Adverse Events and Related Circumstances Captured by the QSRS System Selected for Study **eTable 1.** Observed Occurrence Rates of Individual Adverse Events per 1000 Discharges **eTable 2.** Adjusted Risk of Adverse Events per 1000 Admissions Associated With a 10% Increase in COVID-19 Burden **eFigure.** Adverse Events per 1000 Admissions Associated With Decile of COVID-19 Burden

Supplement 2.
Data Sharing Statement
