# Supplementary material for: Hospital COVID-19 Burden and Adverse Event Rates
Source: JAMA Netw Open. 2024 Nov 4;7(11):e2442936. doi: 10.1001/jamanetworkopen.2024.42936 (PMC11581512; doi:10.1001/jamanetworkopen.2024.42936)
Supplement: Supplement 1. — eBox. Adverse Events and Related Circumstances Captured by the QSRS System Selected for Study eTable 1. Observed Occurrence Rates of Individual Adverse Events per 1000 Discharges eTable 2. Adjusted Risk of Adverse Events per 1000 Admissions Associated With a 10% Increase in COVID-19 Burden eFigure. Adverse Events per 1000 Admissions Associated With Decile of COVID-19 Burden [file jamanetwopen-e2442936-s001.pdf]

Supplementary Online Content

Metersky ML, Rodrick D, Ho SY, et al. Hospital COVID-19 burden and adverse event rates. *JAMA Netw Open*. 2024;7(11):e2442936. doi:10.1001/jamanetworkopen.2024.42936

eBox. Adverse Events and Related Circumstances Captured by the QSRS System Selected for Study

eTable 1. Observed Occurrence Rates of Individual Adverse Events per 1000 Discharges

eTable 2. Adjusted Risk of Adverse Events per 1000 Admissions Associated With a 10% Increase in COVID-19 Burden

eFigure. Adverse Events per 1000 Admissions Associated With Decile of COVID-19 Burden

This supplementary material has been provided by the authors to give readers additional information about their work.

© 2024 Metersky ML et al. *JAMA Network Open*.

eBox. Adverse Events and Related Circumstances Captured by the QSRS System Selected for Study

Measure	Module
Patient sustained one or more adverse outcomes related to receipt of blood or blood product transfusion	Blood and Blood Product
Patients with one or more falls during stay	Fall
Patients sustained one or more maternal adverse outcomes	Birth - Maternal
Adverse event associated with intravenous unfractionated heparin	Medication
Adverse event associated with low molecular weight heparin, thrombin inhibitor or factor Xa inhibitor	Medication
Adverse event associated with warfarin	Medication
Adverse event associated with hypoglycemic agent	Medication
Adverse event within 24 hrs. following opioid administration	Medication
Anaphylaxis	Medication
Possible overdose	Medication
Catheter-associated urinary tract infection (CAUTI)	Healthcare-associated Infection
Clostridioides difficile infection (CDI)	Healthcare-associated Infection
Central line-associated bloodstream infections (CLABSI)	Healthcare-associated Infection
Coronavirus (COVID-19)	Healthcare-associated Infection
Urinary tract infections (UTI)	Healthcare-associated Infection
Hospital - acquired pneumonia not related to surgical procedure (HAP)	Healthcare-associated Infection
Major surgical procedure preceded hospital - acquired pneumonia (HAP)	Healthcare-associated Infection
Surgical site infection following operating room procedure (SSI)	Healthcare-associated Infection
Stays with one or more pressure ulcer adverse events	Pressure Ulcer
Patient sustained one or more adverse outcomes from one or more operating room procedures or instance of anesthesia during stay	Surgery or Anesthesia

© 2024 Metersky ML et al. *JAMA Network Open*.

Measure	Module
Patient developed deep vein thrombosis/pulmonary embolism not related to procedure	Venous Thromboembolism
Patient developed deep vein thrombosis/ pulmonary embolism following listed procedure (no venous thromboembolism prior to procedure)	Venous Thromboembolism
Iatrogenic pneumothorax	Other Outcomes of Interest
Unintended laceration or puncture	Other Outcomes of Interest
Adverse outcome from arterial puncture	Other Outcomes of Interest
Mechanical adverse event associated with central venous catheter	Other Outcomes of Interest
Patient experienced burn/shock during stay (outside of operating room)	Other Outcomes of Interest
Patient attempted suicide during stay	Other Outcomes of Interest
Patient harmed from use of physical restraint (other than bedrails)	Other Outcomes of Interest
Patient harmed from accident associated with bedrails (other than fall)	Other Outcomes of Interest
Intravascular air embolism during stay	Other Outcomes of Interest
Patient elopement during stay	Other Outcomes of Interest

© 2024 Metersky ML et al. *JAMA Network Open*.

eTable 1. Observed Occurrence Rates of Individual Adverse Events per 1000 Discharges

	All Admissions (N=40737)		Principal diagnosis or Secondary diagnosis of COVID-19			
			Yes (N=4114)		No (N=36623)	
**Measure**	**# AEs**	**# AEs rate per 1000 discharges**	**# AEs**	**# AEs rate per 1000 discharges**	**# AEs**	**# AEs rate per 1000 discharges**
Patient sustained one or more adverse outcomes related to receipt of blood or blood product transfusion	63	1.55	7	1.70	56	1.53
Patients sustained one or more maternal adverse outcomes	2	0.05	0	0.00	2	0.05
Patients with one or more falls during stay	262	6.43	48	11.67	214	5.84
Urinary Tract Infections (UTI)	36	0.88	4	0.97	32	0.87
Catheter-associated urinary tract infection (CAUTI)	30	0.74	6	1.46	24	0.66
Central Line-Associated Bloodstream Infections	70	1.72	15	3.65	55	1.50
Clostridium Difficile Infection (CDI) acquired during stay	65	1.60	5	1.22	60	1.64
Covid	15	0.37	14	3.40	1	0.03
HAP (Hospital - Acquired Pneumonia) not preceded by surgery	234	5.74	44	10.70	190	5.19
Major surgical procedure preceded HAP (Hospital - Acquired Pneumonia)	60	1.47	4	0.97	56	1.53
Surgical Site Infection (SSI) following operating room procedures	18	0.44	0	0.00	18	0.49

© 2024 Metersky ML et al. *JAMA Network Open*.

Adverse Event Associated with IV Unfractionated Heparin	113	2.77	22	5.35	91	2.48
Adverse event associated with warfarin	20	0.49	4	0.97	16	0.44
Adverse event associated with LMWH, thrombin inhibitor or factor Xa inhibitor	94	2.31	34	8.26	60	1.64
Adverse Event Associated with Hypoglycemic Agent	636	15.61	121	29.41	515	14.06
Adverse event within 24 hrs. following opioid administration	68	1.67	4	0.97	64	1.75
Anaphylaxis	5	0.12	0	0.00	5	0.14
Possible overdose	1	0.02	0	0.00	1	0.03
Stays with one or more pressure ulcer adverse events	1007	24.72	184	44.73	823	22.47
Patient sustained one or more adverse outcomes from one or more operating room procedures	156	3.83	13	3.16	143	3.90
Other VTEs (not surgery-related)	102	2.50	25	6.08	77	2.10
Patient developed VTE/PE following listed procedure (no VTE prior to procedure)	27	0.66	2	0.49	25	0.68
Iatrogenic pneumothorax associated with Non-OR Procedure	6	0.15	0	0.00	6	0.16
Unintended laceration or puncture associated with Non-OR Procedure	3	0.07	0	0.00	3	0.08
Intravascular air embolism during stay	1	0.02	1	0.24	0	0.00
Arterial puncture during stay	26	0.64	3	0.73	23	0.63
Mechanical adverse event associated with central venous catheter	16	0.39	5	1.22	11	0.30
Patient experienced burn during stay	1	0.02	0	0.00	1	0.03
Patient attempted suicide during stay	1	0.02	0	0.00	1	0.03

© 2024 Metersky ML et al. *JAMA Network Open*.

Patient harmed from use of physical restraint (other than bedrails)	4	0.10	1	0.24	3	0.08
Patient harmed from accident associated with bedrails (other than fall)	3	0.07	2	0.49	1	0.03
Patient elopement during stay	17	0.42	2	0.49	15	0.41

© 2024 Metersky ML et al. *JAMA Network Open*.

eTable 2. Adjusted Risk of Adverse Events per 1000 Admissions Associated with a 10% Increase in COVID-19 Burden[a]

	**All Patients (N=40737)**				**Non-Covid-19 Patients (N=36623)**				**Covid-19 Patients (N=4114)**			
	**Unadjusted**		**Adjusted[b]**		**Unadjusted**		**Adjusted[b]**		**Unadjusted**		**Adjusted[b]**	
	RR (95% CI)	*p*-value	RR (95% CI)	*p*-value	RR (95% CI)	*p*-value	RR (95% CI)	*p*-value	RR (95% CI)	*p*-value	RR (95% CI)	*p*-value
**All Adverse events**	1.16 (1.12-1.2)	<0.001	1.09 (1.05-1.13)	<.001	1.09 (1.04-1.14)	<0.001	1.08 (1.03-1.14)	0.001	1.08 (1.01-1.15)	0.018	1.11 (1.03-1.18)	0.003
**Medication-related adverse events**	1.21 (1.15-1.27)	<0.001	1.11 (1.05-1.17)	<.001	1.13 (1.05-1.21)	0.001	1.1 (1.02-1.19)	0.013	1.14 (1.05-1.24)	0.002	1.17 (1.06-1.29)	0.001
**Hospital-aquired infections**	1.19 (1.11-1.27)	<0.001	1.14 (1.05-1.24)	0.002	1.13 (1.03-1.24)	0.010	1.14 (1.02-1.26)	0.016	1.13 (0.99-1.28)	0.073	1.2 (1.05-1.37)	0.006
**Fall or pressure Ulcer**	1.14 (1.09-1.19)	<0.001	1.07 (1.01-1.13)	0.013	1.07 (1.01-1.14)	0.029	1.07 (1-1.15)	0.040	1.04 (0.95-1.15)	0.358	1.06 (0.96-1.18)	0.229

[a] COVID-19 burden defined as the daily average number of COVID-19 inpatients each week per 100 hospital beds

[b] Adjusted for age, gender, race, admission source, admission urgency, payer source, COVID-19 admission, individual Elixhauser comorbidities, CCS diagnosis code, hospital characteristics and region .

© 2024 Metersky ML et al. *JAMA Network Open*.

eFigure. Adverse Events per 1000 Admissions Associated with decile of COVID-19 Burden[a,b]

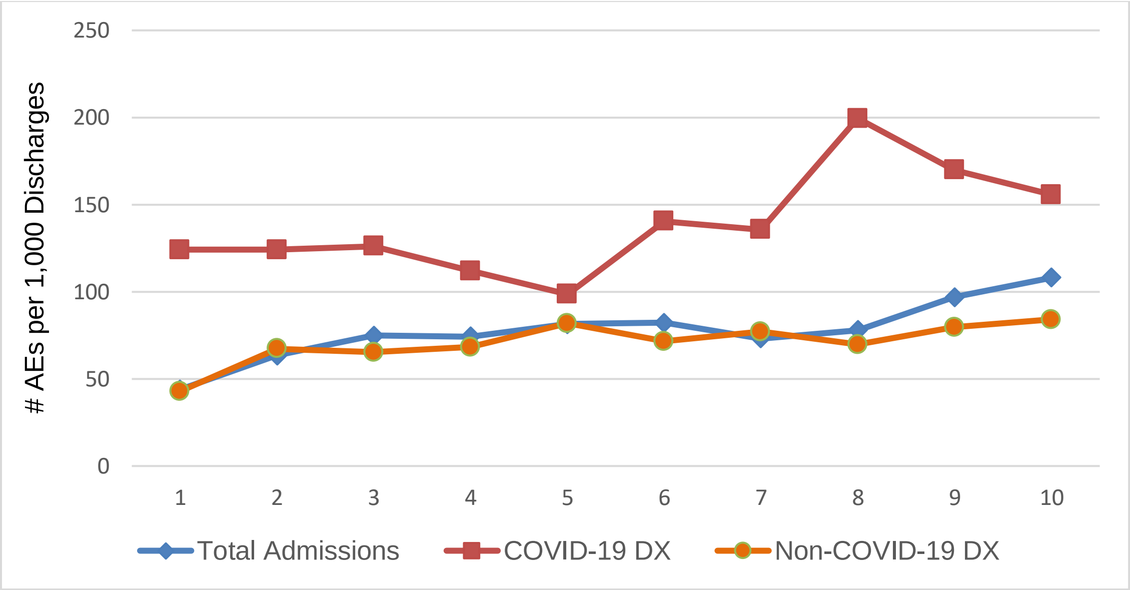

[a] COVID-19 burden defined as the daily average number of COVID-19 inpatients each week per 100 hospital beds

[b]Mean COVID burden for each decile for all admissions: Decile 1-0.02, Decile 2-0.7, Decile 3-1.6, Decile 4-2.6, Decile 5-3.7, Decile 6-5.2, Decile 7-7.4, Decile 8-10,4, Decile 9-15.6, Decile 10-29.8

© 2024 Metersky ML et al. *JAMA Network Open*.
