# Supplementary material for: Hospital COVID-19 Burden and Adverse Event Rates
Source: JAMA Netw Open. 2024 Nov 4;7(11):e2442936. doi: 10.1001/jamanetworkopen.2024.42936 (PMC11581512; doi:10.1001/jamanetworkopen.2024.42936)
Supplement: Supplement 2. — Data Sharing Statement [file jamanetwopen-e2442936-s002.pdf]

Data Sharing Statement

Metersky. Hospital COVID-19 Burden and Adverse Event Rates. *JAMA Netw Open*. Published November 04, 2024. doi:10.1001/jamanetworkopen.2024.42936

Data
Data available: No

Additional Information
Explanation for why data not available: Data use agreement does not allow data to be shared.
